# Potential role of camel, mare milk, and their products in inflammatory rheumatic diseases

**DOI:** 10.1007/s00296-023-05516-x

**Published:** 2024-01-06

**Authors:** Emine Kocyigit, Ruslan Abdurakhmanov, Burhan Fatih Kocyigit

**Affiliations:** 1 Department of Nutrition and Dietetics, Faculty of Health Sciences, Ordu University, 52200 Ordu, Turkey; 2 Department of Biology and Biochemistry, South Kazakhstan Medical Academy, Shymkent, Kazakhstan; 3 Department of Physical Medicine and Rehabilitation, University of Health Sciences, Adana Health Practice and Research Center, Adana, Turkey

**Keywords:** Camel milk, Mare milk, Milk products, Health benefits, Rheumatic diseases

## Abstract

Milk and dairy products serve as a significant dietary component for people all over the world. Milk is a source of essential nutrients such as carbohydrates, protein, fats, and water that support newborns' growth, development, and physiological processes. Milk contains various essential biological compounds that contribute to overall health and well-being. These compounds are crucial in immune system regulation, bone health, and gut microbiota. Milk and dairy products are primarily from cows, buffalos, goats, and sheep. Recently, there has been a notable increase in camel and mare milk consumption and its associated products due to an increasing attraction to ethnic cuisines and a greater awareness of food biodiversity. Camel and mare milk possess diverse nutritional and therapeutic properties, displaying potential functional foods. Camel milk has been linked to various health advantages, encompassing antihypertensive, antidiabetic, antiallergic, anticarcinogenic, antioxidant, and immunomodulatory properties. Camel milk has exhibited notable efficacy in mitigating inflammation and oxidative stress, potentially offering therapeutic benefits for inflammatory disorders. Nevertheless, although extensively recorded, the potential health benefits of mare's milk have yet to be investigated, including its impact on inflammatory conditions. This article highlights the therapeutic potential of camel and mare milk and its derived products in treating inflammatory rheumatic disorders, specifically focusing on their anti-inflammatory and immune-regulatory capabilities. These alternative types of milk, which do not come from cows, offer potential avenues for investigating innovative strategies to regulate and reduce inflammatory conditions.

## Introduction

Milk and dairy products have an important role in human nutrition. In addition to including all the essential nutrients (protein, carbohydrates, fat, and water) for growth and development, it also ensures the preservation of optimal body homeostasis and health improvement [1]. There is a growing global demand for milk and dairy products, particularly in regions such as India, Pakistan, and Africa, where population growth is expected to result in higher consumption of dairy products. Global milk production for 2023 is expected to be approximately 944 million metric tons, with an annual rise of 0.9 percent. Global milk production predominantly originates from cow milk, accounting for around 81% of the total output [2]. The remaining proportion is primarily sourced from buffaloes, goats, and sheep. Less than 1% of the remaining milk supply is derived from camels, yaks, horses, and donkeys [3].

Research on dairy products has historically been centered on cow's milk. However, as population growth, increased milk consumption, and advances in milk technology have occurred, there is a growing need for and interest in non-cow dairy products. The growing interest in milk alternatives, such as camel and mare milk, is closely associated with the geographical areas in which they are reared. Camel, mare milk, and other non-cow dairy products support milk production in unfavorable climatic conditions. Moreover, the consumption of non-bovine milk is linked to the traditions of society. It reflects cultural norms such as consuming fermented mare milk, often known as koumiss, in Central Asia and the intake of camel milk among Bedouin communities. The production of non-bovine milk, which an estimated 150 million people produce, offers these regions a reliable source of food security and a substantial source of income for their families. As a result, milk production from non-cows is crucial in certain nations; it even accounts for a significant amount of total milk production [4–7]. According to a report published in 2019, non-bovine milk production has reached an annual volume of 133 million tons, which accounts for over 17% of the total milk production [8]. The observed rise in milk production rate among small mammal groups has been presented as evidence indicating the presence of market demand and accessibility for non-bovine dairy products [9].

Non-cow milk is rich in oligosaccharides, lipids, biologically active peptides, high-quality protein, minerals, and vitamins, among other substances with benefits for health and nutrition [10]. The therapeutic properties of camel milk include various possible benefits for treating many pathophysiological disorders. The bioactive components of camel milk, including vitamins C, A, and B2, have been associated with various health benefits such as antihypertensive, antidiabetic, antiallergic, anticarcinogenic, antioxidant, and immunomodulatory properties [11, 12]. Some inflammatory disorders, including hepatitis, allergies, lactose intolerance, and alcohol-induced liver damage, have been found to benefit from the use of components found in camel milk, including lysozyme, lactoferrin, and lactoperoxidase. Moreover, antibodies derived from camel milk may help treat inflammatory diseases [13, 14]. Mare milk has been used to treat tuberculosis, gastric ulcers, and chronic hepatitis [15]. The high concentrations of lactoferrin, lysozyme, and immunoglobulins in mare's milk provide a protective mechanism against pathogens, contributing to the body's immune system response. In addition to its enzymatic action, lysozyme exhibits several features, including anti-inflammatory, antiviral, immunostimulatory, antitumor, and anticancer effects [16]. The growing demand and product diversity of camel and mare milk can be attributed to its higher nutritional components, technical advantages, and medical purposes.

There is an absence of studies examining the association between non-bovine milk, particularly camel and mare milk and their products, and inflammatory rheumatic diseases. This study aims to assess the chemical composition of camel milk, mare milk, its derivatives, and their potential in pharmaceuticals. Additionally, this review aims to explore the connection between these substances and inflammation, particularly in the context of inflammatory rheumatic disorders, while highlighting their anti-inflammatory properties.

## Aim of the article

A limited number of studies examine the association between camel and mare milk and their products, which fall under the umbrella of non-bovine milk and inflammation. This article examines the relationship between camel milk, mare milk, and the products derived from these milks in relation to inflammation. It also presents current information on the therapeutic effects of these milks in treating inflammatory rheumatic diseases.

### Search strategy

An extensive search strategy was developed before the literature review, following the methodological approaches suggested by Gasparyan et al. [17]. The search phrase combinations were recognized at the preliminary stage of the study. The search terms that have been provided are as follows: ‘Camel Milk and Rheumatology’ or ‘Camel Milk and Rheumatic Diseases’ or ‘Camel Milk and Inflammation’ or ‘Camel Milk and Health Benefits’ or ‘Camel Milk and Arthritis’ or ‘Camel Milk Products and Rheumatology’ or ‘Camel Milk Products and Rheumatic Diseases’ or ‘Camel Milk Products and Inflammation’ or ‘Camel Milk Products and Arthritis’ or ‘Mare Milk and Rheumatology’ or ‘Mare Milk and Rheumatic Diseases’ or ‘Mare Milk and Inflammation’ or ‘Mare Milk and Health Benefits’ or ‘Mare Milk and Arthritis’ or ‘Mare Milk Products and Rheumatology’ or ‘Mare Milk Products and Rheumatic Diseases’ or ‘Mare Milk Products and Inflammation’ or ‘Mare Milk Products and Arthritis’ ‘Non-Bovine Milk and Rheumatology’ or ‘Non-Bovine Milk and Rheumatic Diseases’ or ‘Non-Bovinel Milk and Inflammation’ or ‘Non-Bovine Milk and Health Benefits’ or Non-Bovine Milk and Arthritis’. Publications from Web of Science, Scopus, PubMed/MEDLINE, and the Directory of Open Access Journals (DOAJ) were included in the study. This study provides an in-depth review of controlled clinical trials, observational studies, reviews, books and book chapters, and scientific papers published in English. The search strategy was not limited to a specific date range; nonetheless, attempts were made to prioritize including more recent articles.

### The physical and chemical composition of camel milk

The global camel population is currently estimated to be 35 million. Out of this total, one million are Bactrian camels with two humps, while the rest are dromedary camels with one hump. Africa is the leading global producer of camel milk. Camel milk is essential to agriculture and food safety in the desert parts of India's subcontinent, Central Asia (including Somalia, Chad, Sudan, Ethiopia, and Kenya), and Africa [18]. Due to the possible health benefits, camel milk is becoming increasingly popular among customers in America and Europe [19, 20].

Camels are often reared in nations characterized by arid regions since a hot temperature, limited water resources, and inadequate grazing land create favorable climatic circumstances for these animals. They find use in several sectors, notably dairy production, transportation, tourism, meat processing, wool production, agriculture, and cosmetics [21]. Camel milk is widely utilized for therapeutic purposes in many cultures, particularly in Asian and African countries, in addition to being consumed as food [18]. The nutritional composition of camel milk is similar to human milk, which has a higher nutritional value than bovine milk. Camel milk is a suitable alternative in cases where there is limited consumption of human milk during infancy. Camel milk has well-balanced essential amino acids and lacks the presence of β-lactoglobulin, a known allergen. Furthermore, it contains essential vitamins, minerals, and bioactive proteins [18, 22]. Camel milk exhibits distinctive properties and demonstrates potential in various areas such as anticancer [23], antidiabetic [24], antibacterial [25], and anti-inflammatory activities [26].

Camel milk exhibits an opaque white color, possesses a slightly acidic pH range of 6.2–6.5, and is characterized by a distinct salty flavor. These attributes change according to the kinds of feed available in the grazing area and water availability for drinking [27]. Camel milk's total protein content ranges from 2.15% to 4.90% [28]. Casein is the predominant protein found in camel milk, accounting for around 52–87% of the total protein content. Specifically, the distribution of casein in camel milk is as follows: β-casein is the most abundant, followed by α-casein, then κ-casein [29]. Whey proteins comprise around 20–25% of the total protein found in camel milk. Among the two primary whey proteins, α-lactalbumin is the predominant constituent in camel milk, whereas β-lactoglobulin is notably deficient. The known antiallergic characteristics of camel milk can be attributed to the lack of β-lactoglobulin, the primary protein associated with bovine milk allergy. Additional whey proteins found in camel milk include peptidoglycan recognition protein, immunoglobulins, serum albumin, and lactoferrin, which are well-known for their antiviral, antibacterial, and immunological characteristics. Camel milk was found to have higher levels of Nacetyl-β-glucosaminidase, lactoperoxidase, lactoferrin, and lysozyme than bovine milk. The casein concentration of camel milk and cow milk exhibits a notable similarity, although the whey protein percentage in camel milk is significantly higher [28, 30].

The fat content of camel milk ranges from 1 to 6% [31]. The primary constituent of camel milk oil is triglyceride, with a relatively low cholesterol concentration. The lipid composition and cholesterol level of camel milk exhibit variability based on factors such as the timing of milking, humidity level, temperature, nutritional factors, lactation stage, and genetic variations among the camel species. The fatty acid composition of camel milk has a small amount of short-chain fatty acids (C4-C12) and a significant amount of long-chain polyunsaturated fatty acids (C14, C16, and C18). The fat globules of camel milk are smaller than those in bovine milk, resulting in enhanced fat digestibility. Besides, the camel milk lipid globule membrane has a high concentration of phospholipids. Camel milk is characterized by its significant content of phospholipid fatty acids and long-chain polyunsaturated fatty acids [32, 33].

Lactose is the primary carbohydrate component in camel milk. Camel milk exhibits similar lactose content to bovine milk; nevertheless, individuals diagnosed with lactose intolerance experience fewer symptoms after consuming camel milk, which can be attributed to its higher metabolic capabilities [34, 35]. Minerals and vitamins may be found in abundance in camel milk. Most minerals are calcium, sodium, magnesium chloride, phosphates, and citrates. The content of vitamin A, vitamin E, thiamine, riboflavin, folic acid, and pantothenic acid in camel milk is lower compared to cow milk. However, the content of pyridoxine and vitamin B12 is nearly identical. According to reports, camel milk has a vitamin C content that is three to five times higher than bovine milk [36, 37].

### Camel milk products

Camel milk is commonly consumed in its raw form or after fermenting. Fermentation is a long-standing technique employed in the production of camel milk [38]. Fermented products are utilized in situations where cooling is unavailable. These products offer distinct taste, texture, and aroma, serving purposes such as prolonging the shelf life of milk, enhancing its nutritional value, and potentially providing health benefits. The most popular fermented camel milk products include *laben* (Arab nations), *ititu* and *dhanaan* (Ethiopia), *garris* (Sudan), *suusac* (Kenya), *khoormog* (Mongolia), and *shubat* (Kazakhstan and China). *Zrig* (Mauritania), *Lfrik* (Morocco), and *chal* (Iran and Turkmenistan) are other fermented products made from a combination of camel milk and water. Fermented camel milk products have been shown to have health benefits due to their antioxidant and anti-inflammatory properties [2, 39]. Milk from camels and cows has similar nutritional content. However, the types, amounts, and distribution of milk proteins and lipids differ. The number of camel milk products (cheese, butter, and yogurt) that have been produced remains limited because of challenges found throughout the production and processing stages [4, 40].

### The physical and chemical composition of mare milk

Horses are domesticated animals historically used for transportation, sports, and warfare. Herodotus reported the health benefits of mare's milk in the fifth century. Mare's milk is an important nutrition source in Central Asian countries, including Mongolia, Kazakhstan, Tajikistan, Kyrgyzstan, and the southern regions of the former Soviet Union [41]. In recent years, there has been a growing interest in mare milk among European consumers, especially in France and Germany [42]. In Italy, mare milk is suggested as an alternative to bovine milk for children allergic to cow's milk [43].

Mare's milk is bioactive and exhibits nutritional and therapeutic benefits [44]. Mare's milk's therapeutic and hypoallergenic characteristics can be attributed to its distinct chemical structure. The chemical structure of this substance exhibits notable distinctions from that of bovine milk yet has similarities to human milk, like camel milk's composition. Mare milk has a range of essential components that benefit the human body. These include amino acids, fats, enzymes such as lysozyme and amylase, and minerals like calcium, sodium, potassium, phosphorus, iron, magnesium, copper, iodine, sulfur, cobalt, zinc, and brom. Additionally, mare milk contains vitamins such as A, C, B1, B2, B6, B12, E, β-carotene, and folic acid. In total, mare milk consists of approximately 40 biological components, all of which are present in a balanced amount [45, 46]. In Mongolia, mare milk has been used as a therapeutic agent for treating chronic infectious disorders, liver disease, and ulcers. For several years, mare's milk has been seen in Russia and Mongolia as an effective treatment for tuberculosis. Furthermore, mare milk and/or kumiss, made by fermenting horse milk, have reportedly been used to cure anemia, nephritis, diarrhea, gastritis, and other digestive disorders [16, 41, 47].

Fresh mare's milk is bluish-white, has a neutral pH of 7.0–7.2, and tastes partially tangy and sweet [16]. The average amount of protein in a mare's milk is 2.3%. The protein composition of mare's milk comprises around 50–55% casein and β-lactoglobulin. Cow milk has more significant amounts of whey protein, α-lactalbumin, immunoglobulin, and lysozyme than cow's milk. Human gastrointestinal enzymes digest the proteins in mare's milk faster than those in cow, goat, camel, and human milk. The majority of caseins in mare's milk (78.5%) are β-casein, with α-casein (20.0%) and κ-casein (1.8%) also present. The whey protein derived from mare's milk is composed of several proteins, including 2–19% barbiturate albumin, 25–50% α-lactalbumin, 28–60% β-lactoglobulin, and 4–21% immunoglobulins. Mare's milk contains significant amounts of lysozyme, lactoferrin, and immunoglobulins. Moreover, mare's milk includes enzymes including amylase, catalase, lipase, peroxidase, phosphatase, malate and lactate dehydrogenase, and lactotransferrin that aid in the digestion of proteins and immune function. Protein digestion produces bioactive peptides with various characteristics, including antibacterial, anti-inflammatory, and blood pressure-regulating capabilities [16, 47, 48].

Mare's milk has a low amount of fat [45]. The lipid composition of mare's milk fat is characterized by approximately 80% triglycerides, 9–10% free fatty acids, and 5–19% phospholipids, which protect cell membranes from oxidative phosphorylation. Mare's milk lipids exhibit a considerable proportion of unsaturated fatty acids, comprising approximately 53% of the overall fatty acid composition, similar to human milk. Notably, mare's milk fat exhibits a high content of medium-chain fatty acids. Furthermore, mare milk is a good source of the essential fatty acids linoleic acid (n-6) and alpha-linolenic acid (n-3) [43, 49].

The composition of carbohydrates in mare's milk mainly contains lactose, along with a small amount of monosaccharides and oligosaccharides [50]. Mare's milk has a similar lactose level to human milk. However, it is more significant than cow's milk. Mare's milk has mostly beta-lactose, compared to cow's milk, which contains primarily α-lactose when the lactose structure and total carbohydrate content are considered. In contrast to α-lactose, β-lactose exhibits a slower absorption rate inside the small intestine. This characteristic contributes to its ability to promote the growth of bacteria within the gut microbiota. The presence of β-lactoses in mare's milk has been found to have a regulatory role in the gut microbiota, exhibiting prebiotic characteristics [16, 51, 52]. Mare's milk is a distinctive nutritional resource that contains a variety of essential vitamins, including vitamins A, B1, B6, B12, C, D, E, and K, as well as essential minerals such as iron, copper, magnesium, manganese, zinc, and calcium [15].

### Mare milk products

In Asia, a wide range of fermented and unfermented mare's milk products such as yogurt and curd for children, cheese, lactic and alcoholic beverages (qymyz, kumiss, or koumiss), and fresh milk are commonly consumed, and their popularity is gradually increasing in Europe [53]. Qymyz is a traditional beverage made from mare's milk fermented with lactic acid and alcohol. It smells fragrant, tastes somewhat sour, and has a fine-grained consistency. It is blue milky white in looks. It is commonly consumed in Central Asian regions and specific areas of Russia, China, and Mongolia. Qymyz has traditionally been utilized for its curative properties. There are a range of neurological and digestive system disorders, tuberculosis, cardiovascular, pulmonary, and urinary tract ailments associated with this treatment's potential benefits. Additionally, it is considered to have potential therapeutic applications for cancer, AIDS, herpes, attention deficit hyperactivity disorder, and insomnia. Furthermore, it has a role in regulating the immune system [43, 54]. Table 1 presents the general composition of milk derived from camels, mares, cows, and humans.

**Table 1:** The general composition of milk from camels, mares, cows, and humans (g/100 mL)

	Camel	Mare	Cow	Human
Energy (kcal)	66.1–88.9	49.4	76.2	64.2
Protein (g)	2.4–4.2	1.4–3.2	3.0–3.9	0.9–1.9
Casein (g)	2.21–2.60	0.94–1.36	2.46–2.8	0.24–0.42
Whey (g)	0.59–0.81	0.74–0.91	0.55–0.70	0.62–0.83
Lactose (g)	3.5–5.1	5.6–7.2	4.4–5.6	6.3–7.0
Fat (g)	2.0–6.0	0.3–4.2	3.3–5.4	2.1–4.0
Total saturated fatty acid (%)	47.0–69.9	37.5–55.8	55.7–72.8	39.4–45.0
Total monounsaturated fatty acid (%)	28.1–31.1	18.9–36.2	22.7–30.3	33.2–45.1
Total polyunsaturated fatty acids (%)	1.8–11.1	12.8 − 51.3	2.4–6.3	8.1–19.1
Linoleic acid (g)	0.08–0.09	0.14	0.06	0.37
α-Linolenic acid (g)	0.04–0.08	0.15	0.04	0.03
Cholesterol (mg)	31.3–37.1	5.0–8.8	13.1–31.4	14–20
Calcium (mg)	106	92.9	119.8	27.6
Magnesium (mg)	12	8.1	12.6	3.8
Potassium (mg)	156	87.1	147.9	71.3
Sodium (mg)	69	17.4	49.3	15.9
Iron (mg)	0.26	0.19	0.08	0.2
Zinc (mg)	0.44	0.62	0.62	0.64
Vitamin C (mg)	10.7–14.4	14	1.0	4.1
Vitamin B_12_ (µg)	0.5	0.3	0.4	0.04

*Adapted and modified from Roy et al. [77] and Fantuz et al.[78]

### Camel, mare milk and their products role in inflammatory rheumatic diseases

Inflammatory rheumatic diseases (IRD) encompass a diverse group of autoimmune conditions characterized by chronicity and heterogeneity [55]. IRD is an umbrella term for a wide range of conditions that include disorders of connective tissue (e.g., systemic lupus erythematosus, Sjögren's syndrome, and systemic sclerosis) and inflammatory arthropathies (e.g., psoriatic arthritis, axial spondyloarthritis, rheumatoid arthritis) [56]. The pathogenesis of IRD remains unclear due to its complex and variable character. However, IRD can be identified by continuous inflammation that predominantly impacts the musculoskeletal system and connective tissue. The progress of disease eventually ends in the deterioration of organs, the development of functional impairments, early mortality, and the imposition of economic and social responsibilities [57].

Several studies have demonstrated the positive effects of the components found in camel milk on health. These bioactive components in fresh and fermented camel milk have been recognized as functional foods [35, 58, 59]. The therapeutic potential of lysozyme, lactoferrin, and lactoperoxidase in camel milk has been recognized for their significant role in managing various inflammatory diseases. The therapeutic effects of camel milk antibodies in regulating the immune system have been shown in the context of inflammatory diseases [14]. Lactoferrin, with potent anti-inflammatory properties, is crucial in promoting the maturation and enhancing the functionality of lymphocytes [60]. Tumor necrosis factor-α (TNF-α) is a significant cytokine involved in immunological modulation. Its primary function is to enhance the inflammatory response and induce oxidative stress by promoting the generation of reactive oxygen species, arachidonic acid metabolites, proteases, and some cytokines. The intake of camel milk has been shown to decrease oxidative stress, which is generated due to the immune system's anti-inflammatory response [23]. Camel milk (10 ml/kg) was administered to rats in a kidney damage model, and it regulated renal inflammation by suppressing myeloperoxidase (MPO), interleukin-1b (IL-1b), IL-18, and monocyte chemoattractant protein-1 [61]. Rats with fenpropatrin-induced neurotoxicity were given 2 ml/day of camel milk, which improved their levels of 3,4-dihydroxyphenylalanine and acetylcholinesterase, decreased their levels of nitric oxide, malondialdehyde, MPO, caspase-3, and TNF-α, and increased the levels of IL-10, total antioxidant capacity, and Bcl-2 [62]. The administration of camel milk to rat models with adjuvant-induced arthritis resulted in decreased levels of TNF-α and an elevated level of the anti-inflammatory cytokine IL-10. In an experimental model of rheumatoid arthritis using rats, the anti-inflammatory properties of camel milk were shown following oral administration of camel milk at a dosage of 10 ml/kg for three weeks. This was evidenced by decreased osteoarthritis index, paw edema, and gait score [63]. A study conducted on rats that were given a high-fat diet demonstrated that incorporating fermented camel milk into their diet, along with a combination of probiotic bacteria such as *Bifidobacterium bifidum*, *St. thermophiles*, and *L. acidophilus*, resulted in a significant decrease in the production of TNF-α and C-reactive protein, which is an inflammatory biomarker [64]. Supplementation of camel milk at doses of 50 and 100 mg/kg/day over 14 days had anti-inflammatory properties, reducing inflammation within fibro-vascular tissue. Numerous mechanisms, including vascularization, collagen deposition, and the suppression of pro-inflammatory cytokines like IL-1b, IL-6, IL-17, and TNF-α, helped to achieve this effect [65]. Results from a study on rats investigating camel milk's anti-inflammatory properties suggested that anti-inflammatory diseases might benefit from adjuvant therapy with camel milk (33 ml/kg) [66].

Camel milk has demonstrated the ability to reduce colonic inflammation by modulating the gut microbiota. He et al. found that camel milk's anti-inflammatory qualities might be used to inhibit colonic inflammation in colitis-affected rats [67]. It has been shown that the peptides produced when lactic acid bacteria (*Lactobacillus plantarum* KGL3A) ferment camel milk have antioxidant and anti-inflammatory effects. Camel milk exerted inhibitory effects on the inflammatory response in the colon by suppressing the excessive production of inflammatory cytokines [68]. The consumption of camel milk has been found to promote the proliferation of *Allobaculum*, *Akkermansia*, and *Bifidobacterium*, enhancing the composition and functionality of the gut microbiota. Camel milk promotes the growth of Allobaculum, a bacterium known for its production of short-chain fatty acids. Short-chain fatty acids have anti-inflammatory properties and promote colon health [69].

The increased concentration of active antioxidant molecules, including lactoferrin, bioactive peptides, and vitamin C, is responsible for the anti-inflammatory benefits of camel milk, fermented camel milk, and their products [70].

The therapeutic benefits of mare's milk are widely known in Russia and Western Asia [71]. Mare's milk possesses several health-promoting characteristics, including antibacterial, antifungal, anti-inflammatory, and antiviral abilities. Additionally, it has been seen to provide favorable health outcomes for those afflicted with cardiovascular disorders and diabetes. Furthermore, it is employed for adjunctive reasons in managing anemia, tuberculosis, gastric ulcers, enteric inflammation, chronic hepatitis B, psoriasis, oncological therapy, post-chemotherapy recovery, postoperative recovery, and post-radiation therapy [72, 73]. However, limited research has examined the association between this factor and inflammation and rheumatic diseases.

**Fig. 1:**
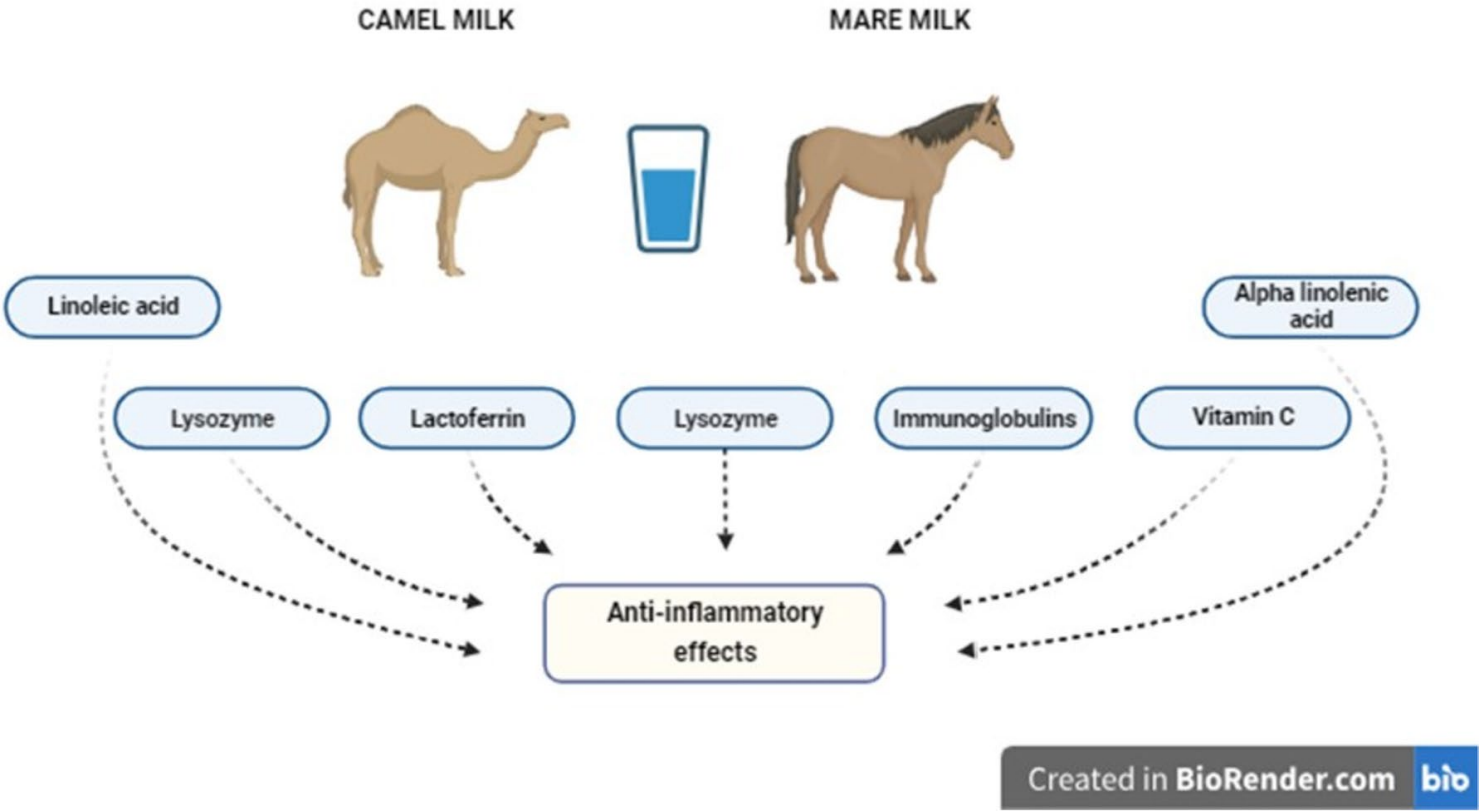
The anti-inflammatory compounds of camel and mare mil. *Adapted and modified from Titisari et al. [76] and Behrouz et al. [79]

The functional parameters of phagocytosis were investigated in 18 healthy participants who consumed 250 ml of mare's milk (either deep-frozen, lyophilized, or cow's milk) daily for three weeks. The study's findings indicate deep-frozen modulates inflammation, reducing chemotaxis and respiratory bursts. This modulation has the potential to provide therapeutic benefits in the treatment of inflammatory diseases [74]. Mare's milk is rich in Lactic Acid Bacteria (LAB), known for its probiotic properties. Lysozyme, immunoglobulins, lactoperoxidase, lactoferrin, and histidine amino acids present in its composition protect against oxidative inflammation. The amount of essential fatty acids in a substance has been found to enhance the absorption of fat-soluble vitamins and regulate inflammatory processes and immune defense mechanisms. Mare's milk also has a higher vitamin C content than cow's milk, hence possessing enhanced nutritional value owing to its antioxidative capabilities and anti-inflammatory characteristics [49, 75, 76]. The anti-inflammatory compounds of camel and mare milk are shown in Fig. 1.

### Limitations

The current literature regarding the efficacy of camel and horse milk and its products in treating inflammatory rheumatic diseases is limited. The association between inflammation, inflammatory rheumatic diseases, camel and horse milk, and its products is not well studied through controlled clinical trials or observational research. The article search was limited to English articles. The limited research in this field may be attributed to the global prevalence of cow's milk consumption, with approximately 85% of individuals worldwide consuming cow's milk and its products. Consequently, research efforts have predominantly focused on investigating the effects of cow's milk consumption.

## Conclusion and future perspectives

Milk and dairy products have historically had significant importance in human nutrition, serving as vital sources of critical nutrients for physical development and overall well-being. The increasing global demand for dairy products has led to a noticeable increase in interest in non-bovine milk, including camel and mare milk. Alternative kinds of milk, characterized by their distinct compositions, have diverse nutritional and possible therapeutic advantages.

Camel milk is notable for its wide range of health-enhancing properties. The existing research suggests it effectively alleviates inflammation, regulates oxidative stress, and modulates immune system function. These qualities open up promising possibilities for managing inflammatory rheumatic disorders and other health issues.

While mare's milk has shown potential as a beneficial substance, limited research has been conducted to investigate its association with inflammatory conditions. However, its nutritious content, including proteins, fats, vitamins, and minerals, implies that it may have yet to discover potential for addressing various health issues.

This article underlines the importance of future research into camel and mare milk use in managing inflammatory rheumatic disorders. Their anti-inflammatory and immune-modulating characteristics provide possibilities for the development of novel approaches. With the ongoing global population growth and the increasing variety of dietary preferences, alternative milk may be a helpful source in enhancing health and well-being.

## Data Availability

There is no data set recorded and stored regarding the article.
